# Case Report: Successful treatment of severe Guillain-Barré syndrome with paralytic ileus as a presenting symptom by intensive immunotherapy

**DOI:** 10.3389/fimmu.2025.1435817

**Published:** 2025-04-02

**Authors:** Long Luo, Lei Chen, Jun Li, Ye Deng, Caihong Chen, Dan Cheng, Yang Liu, Huaping Zhang, Ling Zhu

**Affiliations:** ^1^ Department of Neurology, Xiangtan Central Hospital (The Affiliated Hospital of Hunan University), Xiangtan, Hunan, China; ^2^ Medical Department, Xiangtan Central Hospital (The Affiliated Hospital of Hunan University), Xiangtan, Hunan, China

**Keywords:** ileus, Guillain-Barré syndrome, intensive immunotherapy, case report, GBS

## Abstract

Involvement of the intestinal autonomic nerves in Guillain-Barré syndrome (GBS) can lead to paralytic ileus, a condition commonly observed in severe cases during later stages of the disease. Cases with paralytic ileus as a presenting symptom are very rare. We report a case of a 35-year-old male patient who was admitted to the hospital with acute abdominal pain persisting for 12 hours. Abdominal CT suggested small bowel obstruction, for which routine conventional pharmacological treatment were ineffective. Subsequently, the patient presented with multiple sets of cranial nerve paralysis, bilateral symmetrical delayed paralysis, distal limb numbness, respiratory failure, urinary retention, shock, and electrophysiology, suggesting axonal-type multifocal peripheral nerve damage. Notably, blood antiganglioside tests showed IgG positivity for anti-sulfatide antibodies, anti-GD1a antibodies, and anti-GT1a antibodies. The patient was administered plasma exchange combined with intravenous immunoglobulin, and symptoms gradually improved. The patient resumed independent ambulation within two months and returned to normal status at one year, with no recurrence of symptoms. Given that paralytic ileus can precede other neurological abnormalities in patients with GBS, early detection and individualized treatment are critical to reduce the risk of death and promote recovery. Here, we demonstrate that intensive immunotherapy is a viable therapeutic approach that can be clinically adopted for such conditions.

## Introduction

Guillain-Barré syndrome (GBS) is an autoimmune peripheral neuropathy characterized by acute flaccid paralysis. It affects individuals of all age groups worldwide. Despite standard immunotherapies, approximately 5% of those affected succumb to the condition, and an additional 20% are unable to walk independently one year after disease onset (1). Approximately 20% of people with GBS experience respiratory failure requiring mechanical ventilation (2), which poses a severe threat to human health.

Autonomic dysfunction has been reported in more than half of patients with GBS, primarily affecting the cardiovascular system, urinary tract, and intestines. Paralytic ileus is a rare occurrence typically observed in severe cases during the later stages of the disease (3). However, in rare circumstances, paralytic ileus can manifest as the initial and sole presentation of GBS in the early stages of the disease (4). Moreover, patients with GBS who have comorbid autonomic dysfunction experience a higher mortality rate and slower neurological recovery, requiring meticulous medical attention (5, 6), and the treatment of such patients remains challenging, especially in severe cases.

The pathophysiology of GBS is complex, with distinct mechanisms potentially underlying the demyelinating and axonal subtypes. Anti-ganglioside antibodies, particularly anti-GM1 and anti-GD1a antibodies, are closely related to the pathogenesis of the axonal form of GBS (acute motor axonal neuropathy, AMAN; acute motor and sensory axonal neuropathy, AMSAN). Although the pathological hallmark of the demyelinating form of GBS (acute inflammatory demyelinating poly neuropathy, AIDP) is the phagocytosis of myelin by macrophages, the relationship between autoantibodies to AIDP has not been fully clarified (7, 8). Notably, the pathophysiological mechanisms underlying GBS are still being elucidated, with both T cells and B cells likely implicated in its pathogenesis (9). The complexity of the mechanisms indicates that there is room for improvement in therapeutic strategies.

## Case presentation

**Figure 1:**
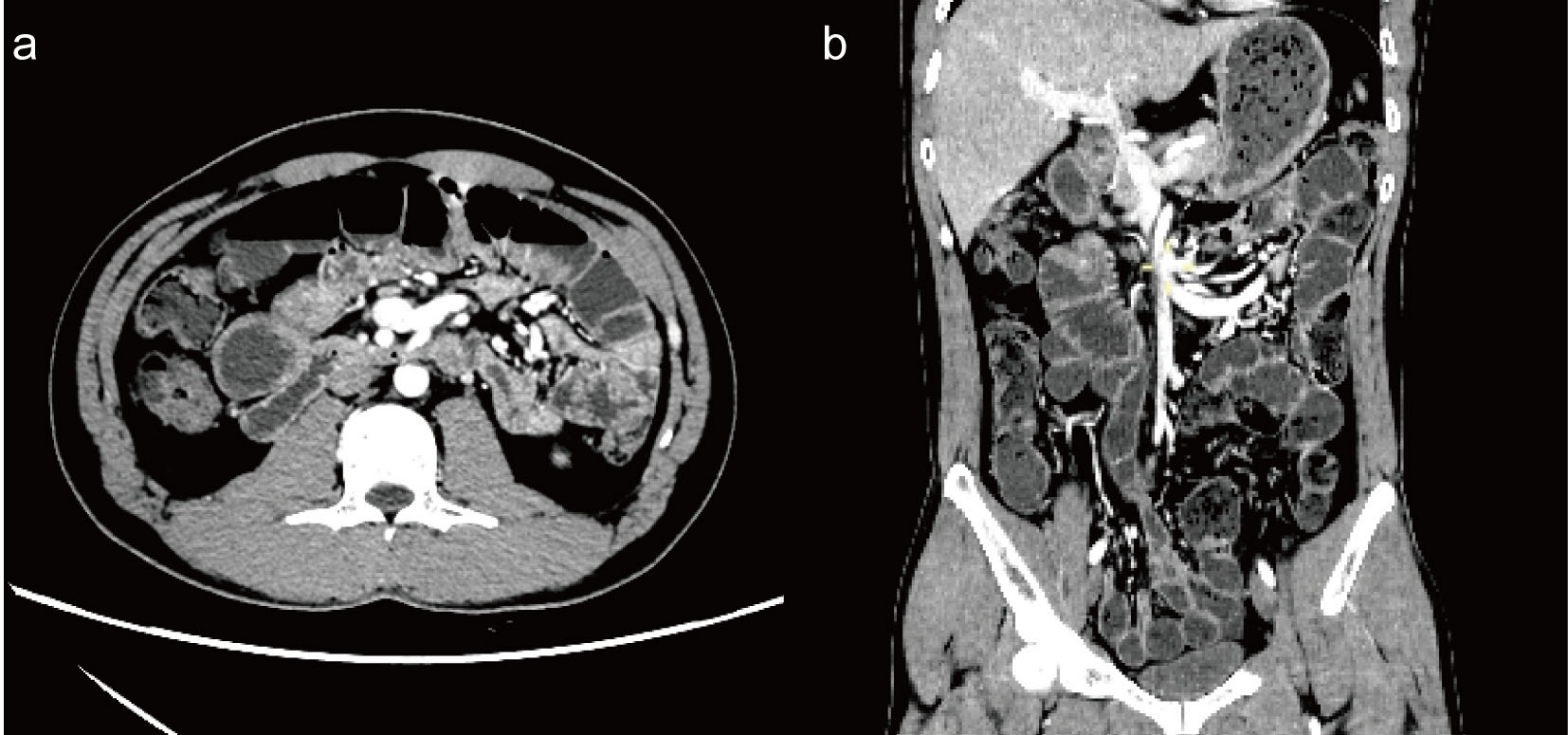
Enhanced CT of the abdomen [**(a)** axial view; **(b)** coronal view] shows multiple small bowel and colon bowel dilatations, mild to moderate enhancement of the bowel wall, and multiple air-liquid planes, with no evidence of obstruction points.

A 35-year-old man was admitted to the emergency department with abdominal pain and distension for 12 hours without other clinical manifestations. The patient was transferred to the general surgery department, where abdominal CT suggested a small bowel obstruction (**Figure 1**). Upon examination, he had a temperature of 36.7°C, heart rate of 85 bpm, blood pressure of 125/75 mmHg, and respiratory rate of 12 breaths/min. The patient’s abdomen was slightly distended, and pressure pain was noted around the umbilicus, with no significant rebound pain, and diminished bowel sounds. The electrocardiogram was normal, and no significant abnormalities were found in arterial blood gas analysis, complete blood count, coagulation profile, D-dimer, liver and kidney function tests, electrolytes, cardiac enzymes, or tumor markers, revealing no evidence of electrolyte imbalances, inflammatory acute abdomen (such as appendicitis, cholecystitis, or pancreatitis), intestinal mucosal ischemia or necrosis, or intra-abdominal infections—common causes of ileus, and the patient’s ileus showed no response to treatments such as paraffin oil enema and catharsis, gastrointestinal decompression, antibiotics, proton pump inhibitors, or somatostatin therapy. The patient denied a history of antecedent infection, and the other medical histories were unremarkable. On the third day of admission, the patient had bilateral eyelid ptosis, hoarseness, limited tongue extension, neck weakness, a Medical Research Council of the Extremities (MRC) grade of 4/5, and a negative Babinski’s sign. No significant abnormalities on the brain MRI scan were observed on day 3, and the etiology of the patient’s symptoms remained undetermined. On the fourth day of admission, the patient experienced the loss of deep tendon reflection in all four limbs, with an MRC grade of 2/5, accompanied by loss of tactile and pain sensation in the distal limbs, respiratory failure requiring mechanical ventilation, shock requiring pressurization, and urinary retention requiring catheterization. After an urgent multidisciplinary consultation, the patient was clinically diagnosed with GBS with an mEGOS score of 9 (10) and transferred to the Neuro ICU for plasma exchange (PE) (40 ml/kg/qod). A lumbar puncture was performed on the same day, revealing that the cerebrospinal fluid routine and biochemical tests were within normal ranges.

The patient’s condition did not improve after receiving the initial PE, and it continued to progress. On the sixth day of admission, the patient became lethargic, was unable to open his eyes, had fixation of both eyeballs, and had an MRC grade of 1/5 in the extremities and a Hughes score of 5. Electromyography (EMG) performed on day 6 suggested bilateral symmetrical axonal-type motor-sensory poly peripheral neuropathy with a peroneal nerve-sparing pattern, lack of F-waves, disappearance of bilateral blink reflexes, and the EMG results supporting the diagnosis of GBS. The patient then received three additional PEs (40 ml/kg/qod). After four PE sessions, the patient regained alertness, eye movement returned, and limb muscle strength was achieved with an MRC grade of 3/5. However, he consistently had persistent poor gastrointestinal emptying, respiratory weakness, and exposure to life-threatening conditions. The patient was provided a course of intravenous immunoglobulin (IVIG), as it is possible for individual patients to benefit from intensive treatment early in the disease before irreversible neurologic damage occurs.

**Figure 2:**
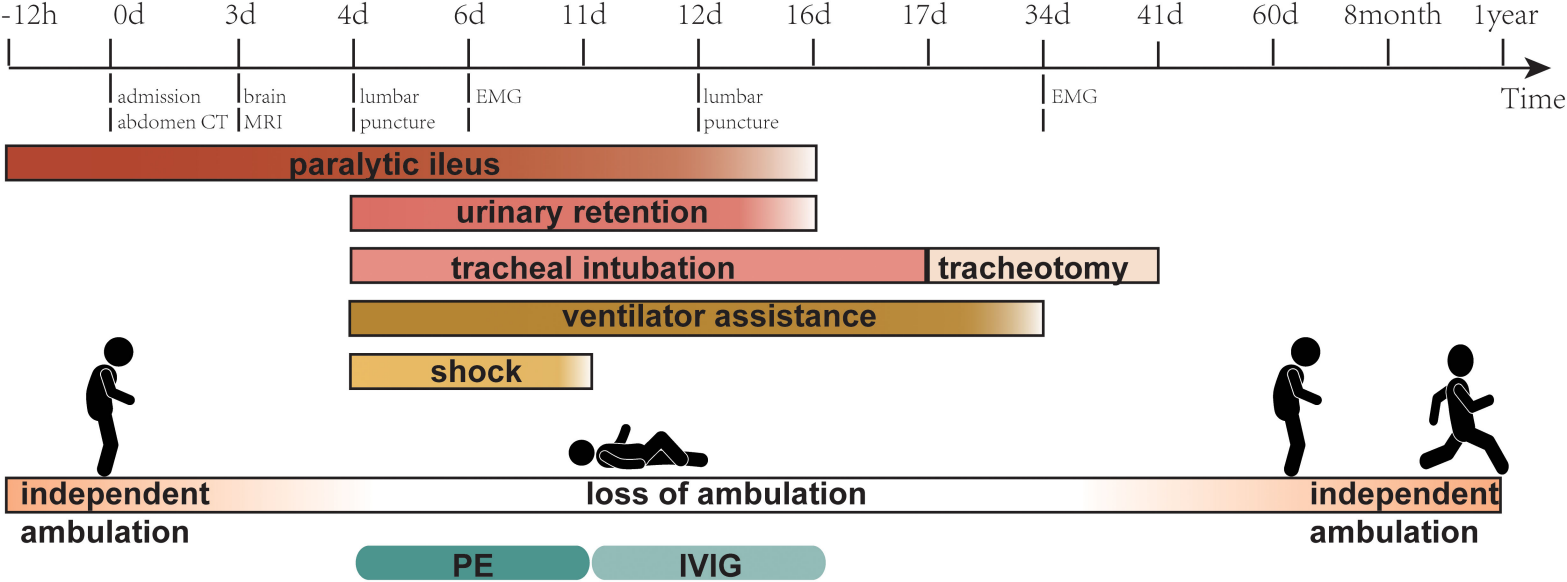
Timeline showing the main relevant events in the case. MRI, magnetic resonance imaging; EMG, electromyogram; PE, plasma exchange; IVIG, intravenous immunoglobulin.

A repeat lumbar puncture was performed on day 12 of admission; the CSF routine and biochemical test findings were still in the normal range. Notably, immunoblotting assays revealed positive results for serum anti-sulfatide IgG, anti-GD1a IgG, and anti-GT1a IgG, whereas cerebrospinal fluid tests were negative. On day 16, the patient resumed urination, anal exhaust, and defecation, and enteral nutrition was administered. Based on the patient’s medical history, auxiliary examinations, and response to treatment, we concluded that the paralytic ileus was of GBS origin. However, the patient’s respiratory function improved slowly and repeated weaning tests failed. On the 17th day of admission, a tracheotomy was performed. By the 34th day, the patient was successfully weaned off the ventilator, and a repeat EMG examination was conducted, revealing that the amplitudes of the compound muscle action potential and sensory nerve action potential were significantly higher than before. Moreover, the F-wave latencies of the bilateral median, ulnar, and tibial nerves were all within normal ranges, with an occurrence rate of 100%. The blink reflex also returned to normal. The tracheostomy tube was removed on the 41st day. He resumed independent walking on the 60th day but required assistance getting up from a squat, which he could perform without assistance at the 8th month. After one year, the patient could run, albeit not as well as before the onset of the disease (**Figure 2**).

## Discussion

Our patient initially presented with symptoms unsuggestive of GBS, such as abdominal pain and distension (11). However, the patient subsequently developed a typical cluster of GBS symptoms, including symmetrical delayed paralysis, numbness of the extremities at the ends, cranial nerve involvement, urinary retention, shock, and respiratory failure. The diagnosis of GBS was confirmed based on electrophysiological studies, the presence of positive anti-ganglionic antibodies, the effectiveness of immunotherapy, the absence of any other explanatory etiologies, and a unidirectional course of the disease.

Ileus occurs in approximately 16% of patients with GBS (3, 12). However, this figure may be overestimated owing to the lack of uniform diagnostic criteria and difficulty distinguishing GBS-induced ileus from confounding factors, such as critical illness, electrolyte disturbances, immobility, and medications (e.g., anesthetics) (3, 13). Paralytic ileus is thought to be caused by GBS damage to the gastrointestinal autonomic nerves. Autopsy results confirm autonomic demyelination is present in patients with GBS (14), resulting from an immune response to the autonomic nerves (15). Spectral analysis of heart rate variability revealed a marked shift in sympathovagal balance to sympathetic dominance at the height of GBS disease (16), and similar alterations have been observed in Miller Fisher syndrome (17). Sympathetic dominance may also be present in the autonomic nervous system of the gastrointestinal tract during the acute phase of GBS, leading to slow gastrointestinal motility and potentially ileus (13).

Gangliosides are widely present in tissue cells and are particularly abundant in nerve cell myelin membranes, axon membranes, neuromuscular junctions, and nodes of Ranvier. Antibody binding to gangliosides in these regions may lead to disturbances in axon-glia interactions, disorders in ion channel regulation, and trigger axonal degeneration (18, 19). This process can destabilize cytoskeletal structures and impede nerve conduction (8, 18). Antibodies against gangliosides are occasionally associated with clinical signs suggestive of selective nerve damage. Our patient tested positive for blood anti-GT1a, anti-GD1a, and anti-sulfatide antibodies. Anti-GT1a antibodies have been associated with somnolence, ataxia, extraocular muscle paralysis, oropharyngeal involvement, medullary paralysis, decreased tendon reflexes, and pharyngeal and cervical brachial weakness (20, 21). Anti-GD1a antibodies have been implicated in the pathogenesis of AMAN and AMSAN and have been associated with distal dominant weakness, neck weakness, and cranial nerve injury (21). Neuropathic manifestations of anti-sulfatide antibodies are highly heterogeneous and have previously been reported to be associated with sensory axonal neuropathies with small fibers, ataxia, and pain (22). Our patient’s clinical presentation is consistent with those reported in previous studies.

The observation that our patient’s condition continued to deteriorate after the initial PE is a commonly observed phenomenon and is considered a natural progression of the disease. At least 25% of patients experience a decline during or shortly after treatment with IVIG or PE. This phenomenon is not attributed to drug resistance, as the patient’s medical status would have continued to worsen in the absence of treatment (23). Following a brief exacerbation of symptoms, the patient gradually experienced recovery.

Although studies in Western countries have shown no significant difference between PE combined with IVIG and PE treatment alone, the sample size in these studies may not have been large enough to rule out a small beneficial effect of combination therapy (24). In addition, previous studies did not include a sufficient number of participants with axonal GBS to determine whether they responded differently to treatment compared to those with AIDP (25). Geography appears to influence the GBS electrophysiologic subtypes, with axonal subtypes being higher in Asian countries than in Europe and the United States of America (26). An observational study based in Japan found that intensive immunotherapy was superior to a single course of Propecia in serious patients (mEGOS ≥ 7 points on admission) (27). However, a randomized controlled trial conducted in the Netherlands demonstrated the ineffectiveness of a second course of IVIG (28). Differences in studies did not exclude correlations with different electrophysiological characteristics, since pathogenic mechanisms of axonal and demyelinating types could differ (7, 8). Moreover, patients with GBS have high heterogeneity, and intensified immunotherapy shows potential benefits for patients with treatment-related fluctuations and severe cases (27, 29, 30). Additionally, the mechanisms of PE and IVIG therapy are distinct. PE functions by removing circulating autoantibodies, whereas IVIG therapy neutralizes autoantibodies, inhibits complement activation, suppresses the formation of membrane attack complexes, and acts by blocking antibody production. According to preliminary evidence, PE combined with IVIG shows significant efficacy in severe pediatric cases, indicating a potential synergistic effect (30). Our patient was classified as AMSAN according to the electrodiagnostic criteria summarized by Uncini (31); his mEGOS score on day 4 of admission was 9. He gradually improved after receiving intensive immunotherapy without any significant side effects. Lee et al. reported a similarly severe case of GBS that started with ileus, followed by severe delayed paralysis and respiratory failure. The patient recovered his muscle strength after one course of IVIG, but poor gastric emptying persisted, and enteral nutritional support was unavailable. He ultimately died from a severe infection (32). In contrast, relatively mild cases of GBS presenting with ileus have been reported to respond well to IVIG (4, 33, 34). Although we do not know the outcome of PE or IVIG treatment alone in our patient, to our knowledge, this is the only reported case of severe GBS starting with paralytic ileus that has been successfully treated. More evidence and trials are required to establish a treatment plan.

## Conclusion

Clinical practitioners should consider the possibility of GBS when encountering acute abdominal pain or paralytic ileus to avoid misdiagnosis or underdiagnosis, especially during non-specialty visits. Early diagnosis and timely individualized treatment are essential to improve patient prognosis.

## Data Availability

The original contributions presented in the study are included in the article/**Supplementary Material**. Further inquiries can be directed to the corresponding author.
