# Supplementary material for: Case Report: Successful treatment of severe Guillain-Barré syndrome with paralytic ileus as a presenting symptom by intensive immunotherapy
Source: Front Immunol. 2025 Apr 2;16:1435817. doi: 10.3389/fimmu.2025.1435817 (PMC12000006; doi:10.3389/fimmu.2025.1435817)
Supplement: Supplementary file 1 [file DataSheet2.doc]

Motor conduction velocity

Motor conduction velocity	latency	amplitude	distance	Conduction velocity	F-wave latency
	ms	mv	mm	m/s	ms
**Left ulnar nerve movement**					
wrist - ADM	2.62	1.62			--
below elbow - wrist	5.83	1.38	165	51.4	
Erb - below elbow	12.3	0.64	310	47.9	
**Right ulnar nerve movement**					
wrist - ADM	2.62	1.78			---
below elbow - wrist	5.84	1.65	165	51.2	
above elbow - below elbow	7.92	1.35	110	52.9	
axil - above elbow	9.62	1.23	90.0	52.9	
Erb- axil	13.9	1.05	230	53.7	
**Left median nerve motor**					
wrist - APB	3.61	1.62			--
elbow - wrist	7.44	1.38	215	56.1	
Erb - elbow	12.7	0.39	280	53.2	
**Right median nerve motor**					
wrist - APB	4.08	3.1			--
elbow - wrist	8.02	2.6	210	55.0	
axil - elbow	10.0	2.1	105	53.0	
Erb- axil	14.0	1.92	230	57.5	
**Left tibial nerve movement**					
Inner malleolus - AH	4.14	5.6			
Popliteal fossa - Inner malleolus	12.9	3.8	395	45.1	
**Right tibial nerve movement**					
Inner malleolus - AH	4.43	6.7			
Popliteal fossa - Inner malleolus	12.9	4.8	395	46.6	
**Left common peroneal nerve movement**					
instep - EDB	5.71	0.61			
Under the fibula - instep	12.7	0.45	330	47.2	
Above the fibula - Under the fibula	14.9	0.30	100	45.5	
**Right common peroneal nerve movement**					
instep - EDB	4.08	0.84			
Under the fibula - instep	11.4	0.73	330	45.1	
Above the fibula - Under the fibula	13.7	0.58	100	43.5	
**Facial nerve motor left**					
Ear - frontalis	not detected	--			
Ear - Orbicularis oculi muscle	not detected	--			
Ear - Orbicularis oris muscle	not detected	--			
Ear - mentalis	not detected	--			

Facial nerve motor right					
Ear - frontalis	not detected	--			
Ear - Orbicularis oculi muscle	not detected	--			
Ear - Orbicularis oris muscle	not detected	--			
Ear - mentalis	not detected	--			

sensory conduction velocity

sensory conduction velocity	latency	amplitude	distance	Conduction velocity
	ms	uv	mm	m/s
**Left ulnar nerve sensation**				
finger V - wrist	2.17	7.8	115	53.0
**Right ulnar nerve sensation**				
finger V - wrist	2.22	4.7	115	51.8
**Left median nerve sensation**				
finger III - wrist	2.46	7.3	130	52.8
**Right median nerve sensation**				
finger I - wrist	2.04	7.5	105	51.5
finger III - wrist	2.53	5.7	135	53.4
**Left sural nerve sensation**				
Achilles' tendon - Lateral malleolus	2.68	13.8	120	44.8
**Right sural nerve sensation**				
Achilles' tendon - Lateral malleolus	1.98	17.3	100	50.5

H reflection

Left tibial nerve HReflex			
	M-Lat	H-Lat	H/M Amp
	ms	ms	-
Popliteal fossa - Gastrocnemius muscle	6.7	30.3	0.32

Right tibial nerve HReflex			
	M-Lat	H-Lat	H/M Amp
	ms	ms	-
Popliteal fossa - Gastrocnemius muscle	4.7	31.0	0.25

F Data and curves

Left ulnar nerve FResponse					
	M-Lat	F-Lat	F-Lat Mean	F-M Lat	F%
	ms	ms	ms	ms	%
wrist - ADM	--	not detected	--	--	--

左尺神经

腕 -ADM
M:5mV/D 5ms/D
F:2mV/D 5ms/D

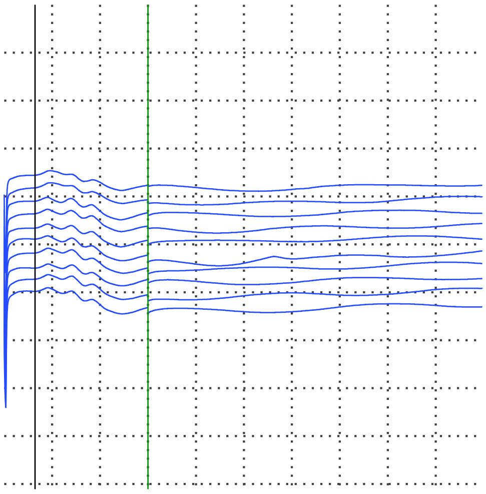

Right ulnar nerve FResponse					
	M-Lat	F-Lat	F-Lat Mean	F-M Lat	F%
	ms	ms	ms	ms	%
wrist - ADM	--	not detected	--	--	--

右尺神经

腕 -ADM
M:5mV/D 8ms/D
F:0.5mV/D 8ms/D

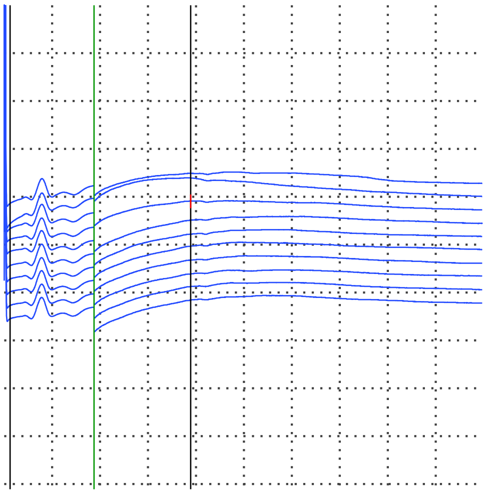

Left median nerve FResponse					
	M-Lat	F-Lat	F-Lat Mean	F-M Lat	F%
	ms	ms	ms	ms	%
wrist - APB	--	not detected	--	--	--

左正中神经

腕 -APB
M:5mV/D 8ms/D
F:1mV/D 8ms/D

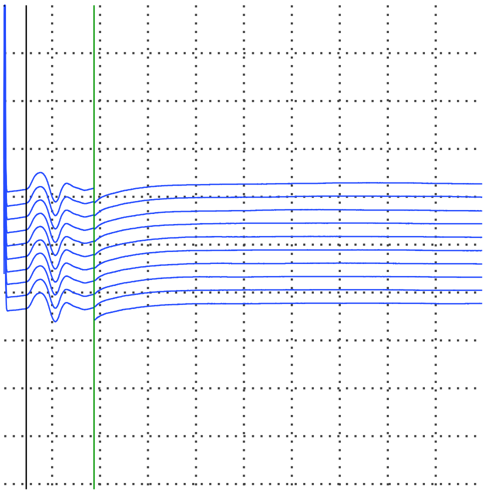

Right median nerve FResponse					
	M-Lat	F-Lat	F-Lat Mean	F-M Lat	F%
	ms	ms	ms	ms	%
wrist - APB	--	not detected	--	--	--

右 正中神经

腕 -APB
M:5mV/D 5ms/D
F:0.5mV/D 5ms/D

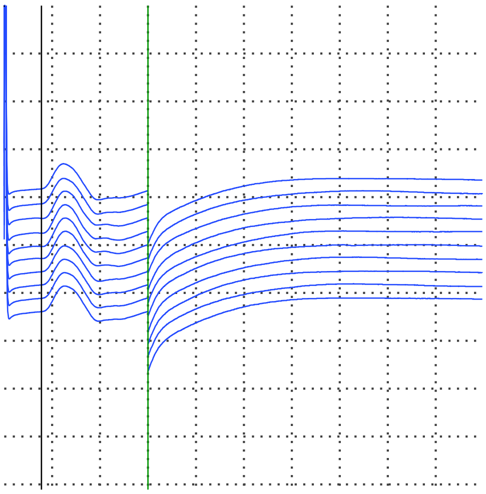

Left tibial nerve FResponse					
	M-Lat	F-Lat	F-Lat Mean	F-M Lat	F%
	ms	ms	ms	ms	%
ankle - AH	--	not detected	--	--	--

左 胫神经

踝 -AH
M:5mV/D 10ms/D
F:2mV/D 10ms/D

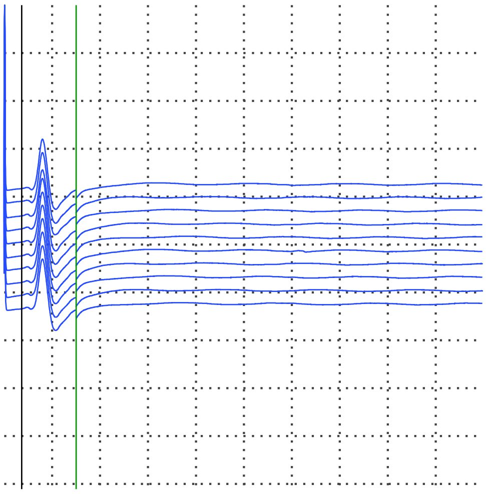

Right tibial nerve FResponse					
	M-Lat	F-Lat	F-Lat Mean	F-M Lat	F%
	ms	ms	ms	ms	%
ankle - AH	4.5	53.5	54.0	--	20

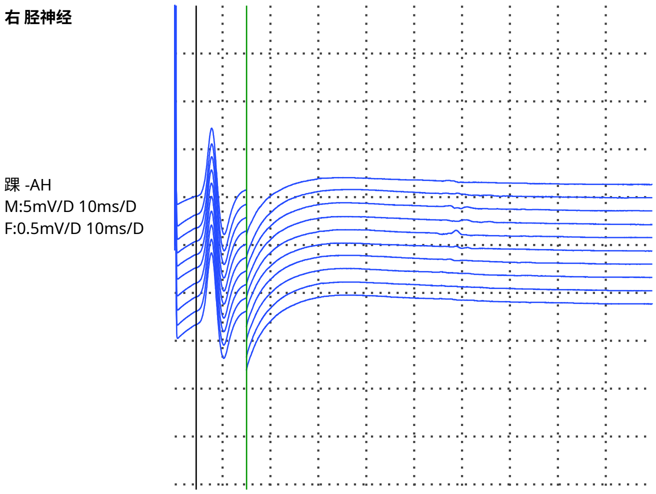

Blink reflex

CC Stim					
stimulate	record	R1 latency		R2 latency	
		[ms]	RefDev	[ms]	RefDev
left	left	not detected		not detected	
	right			not detected	
	difference			-	
right	left	not detected		not detected	
	right			not detected	
	difference			-	
left - right		-		-	
